# Who is following me? Public attitude towards government tracing apps in the covid Era in Israel: Shiran Bord

**DOI:** 10.1093/eurpub/ckac131.224

**Published:** 2022-10-25

**Authors:** O Golan, S Bord, C Satran

**Affiliations:** Health Systems Management, Yezreel Valley College, Yokneam ilit, Israel

## Abstract

**Background:**

In the battle to decrease coronavirus infection and mortality, Israel has employed emergency tools, e.g., tracking civilians’ locations via their cellphones or activating the HaMagen app that identifies when a person is near someone who has been diagnosed with the virus. While the aim of these tools is to ensure the public’s health, they could harm human rights.

**Objective:**

To examine the Israeli public’s attitudes towards enhancing public health during the pandemic while preserving privacy, by examining the relationship between trust in the healthcare system, threat perceptions, cellphone tracking, and HaMagen App.

**Methods:**

Surveys (distributed by iPanel) was completed by 741 adults, aged ≥18.

**Results:**

About half the respondents (47.1%) perceived cellphone tracking as harmful to privacy, yet one-quarter (24.4%) reported that this increases their sense of security. About half (48.4%) agreed/greatly agreed with the item whereby the government uses the gathered data for non-coronavirus purposes. Jewish respondents had more positive attitudes towards government tracking than Arab ones, yet the latter reported higher downloading of HaMagen. The findings indicate that threat perceptions and positive attitudes towards cellphone tracking were related to greater chances of downloading the app. Moreover, attitudes towards such tracking were mediated by the relationship between trust levels/threat perceptions and downloading the app, whereby the former was association with more positive attitudes towards cellphone tracking, which in turn was related to greater app downloading.

**Conclusions and Recommendations:**

Trust plays a central role in people’s willingness to forgo their privacy for the good of public health. To enhance trust, messages must be suited to a range of communities, presented in a suitable language by local professionals.

**Key messages:**

• Trust plays a central role in people’s willingness to forgo their privacy for the good of public health.

• Concern for public health must include ethical considerations.

